# A Minor Dihydropyran Apocarotenoid from Mated Cultures of *Blakeslea trispora*

**DOI:** 10.3390/molecules171112553

**Published:** 2012-10-24

**Authors:** Alejandro F. Barrero, M. Mar Herrador, Pilar Artega, José-Antonio González, Jesús F. Arteaga

**Affiliations:** 1 Departamento de Química Orgánica, Facultad de Ciencias, Universidad de Granada, Avda. Fuente Nueva, s/n, 18071 Granada, Spain; 2 Departamento de Ingeniería Química, Química Fisíca y Químíca Orgánica, Facultad de Ciencias Experimentales, Universidad de Huelva, Campus el Carmen, s/n, 21071 Huelva, Spain

**Keywords:** *Blakeslea trispora*, apocarotenoid, dihydropyrane, isolation, structural elucidation

## Abstract

The heterocyclic C15 apocarotenoid **1** was isolated from mated cultures of the strains F986 (+) and F921 (−) of *Blakeslea trispora*. This new compound formed during sexual interaction is a minor constituent of the culture media and its structure was elucidated by spectroscopic data, including 2D-NMR. A plausible biosynthetic pathway involving a double degradation of β-carotene, followed by several oxidations of the resulting monocyclofarnesane C15 fragment is proposed.

## 1. Introduction

**Figure 1:**
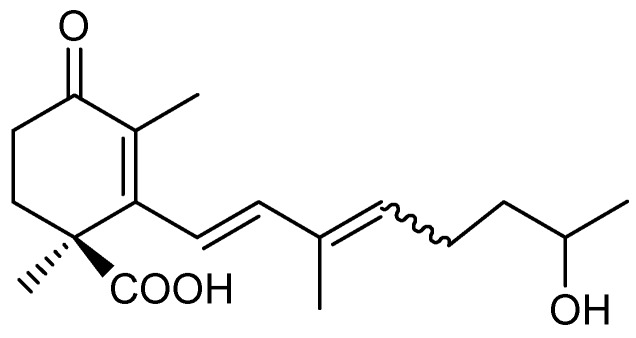
Trisporic C acid (**1**).

*Blakeslea trispora* (syn. *Choanephora trispora*, Mucoromycotina, Mucorales, Choanephoraceae) is used for the industrial preparation of β-carotene, a natural pigment antioxidant and pro-vitamin A with many applications in the food, pharmaceutical, and cosmetic industries [1,2,3,4]. The wild-type strains of this fungus belong to either the (+) or the (–) sex, and many pairs of opposite sex strains, cultured together (“mated” cultures”) increase their β-carotene content and spark the morphological program of the sexual cycle. These physiological effects were attributed to the action of apocarotenoids such as trisporic acid C (**1**, Figure 1) and similar compounds present in mated cultures of *Blakeslea* [5,6,7,8,9,10]. The culture media of *Blakeslea* contains apocarotenoids belonging to the following three families: “trisporoids” with 18 carbons [5,11,12,13,14,15,16], “cyclofarnesoids” with 15 carbons [16,17,18,19,20] and those featuring seven carbons [16]. As a follow-up to our study about production and identification of bioactive apocarotenoids from *Blakeslea trispora*, in this paper we report the isolation and structural elucidation of the minor cyclofarnesane apocarotenoid **2** from mated cultures of *B. trispora*. Additionally a plausible biosynthetic pathway justifying its formation during sexual interaction is presented.

## 2. Results and Discussion

**Figure 2:**
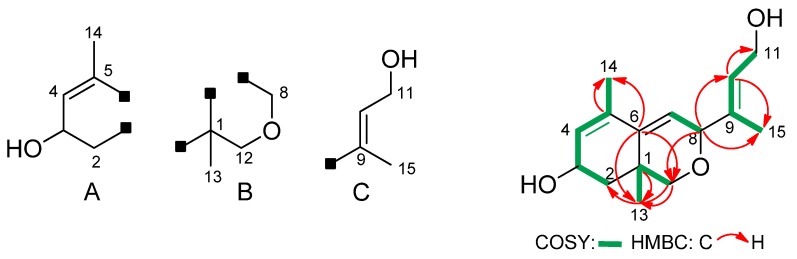
Key COSY and HMBC correlations for apocarotenoid **2**.

Sexually (+) and sexually (−)*B. trispora* strains F921 and F986 were cultured together for three days on agar medium. Following semi-preparative normal phase HPLC separation, a careful search for the neutral metabolites in the agar extracts has permitted the isolation of a few mg of compound **2** (relative concentration: 4 mg in 1 L of medium)**.** It is important to point out that compound **2** is not present in single cultures meaning that the product was produced during sexual interaction. This compound was isolated as a colourless syrup and high-resolution mass spectrum (FAB^+^) showed a molecular ion [M+Na]^+^ at *m/z* 273.1464, corresponding to a molecular formula C_15_H_22_O_3_ (five degrees of unsaturation) and its IR spectrum exhibited an absorption band corresponding to a hydroxyl group (3417 cm^−1^). The ^13^C-NMR and HSQC spectra revealed 15 carbon signals, including three methyl groups, three methylene groups (two oxygenated), five methyne groups (two oxygenated and three sp^2^) and four quaternary carbons (three sp^2^). These data establish the presence of three double bonds, two rings (one oxygenated) and two hydroxyl groups in the structure of **2**. Some of the COSY and HMBC correlations depicted in Figure 2 established the presence of frameworks A–C in its structure. Connectivity among these frameworks was deduced from the HMBC correlations (Figure 2, Table 1).

**Table 1:** Mono- and bi-dimensional NMR data for compound **2**.

C/H	δ_H_	δ_C_		COSY	HSQC	HMBC
1			32.1			
2a	1.65–1.42 m		38.4	H3, H2b	C2	C12a, C13
2b	1.65–1.42 m			H3, H2a		
	4.34 br s		64.4	H4, H2a, H2b	C3	
4	5.72 d (4.6)		125.8	H3, H14	C4	
5			133.0			
6			140.9			
7	5.65 d (3.3)		120.5	H8	C7	
8	4.64 br s		76.5	H7	C8	
9			138.0			
10	5.54 t (6.4)		127.3	H11, H15	C10	C8, C15
11	4.24 d (6.4)		59.5	H10	C11	C9, C10
12a	3.32 d (10.7)		70.5	H12b, H13	C12	C8, C6, C13
12b	3.36 d (10.7)			H12a, H13		
13	1.35 s		24.6	H12a, H12b	C13	C12, C6, C2a, C1
14	1.86 s		19.4	H4	C14	C6, C5, C4
15	1.77 s		14.9	H10	C15	C8, C9, C10

*J* in Hz in parentheses.

**Figure 3:**
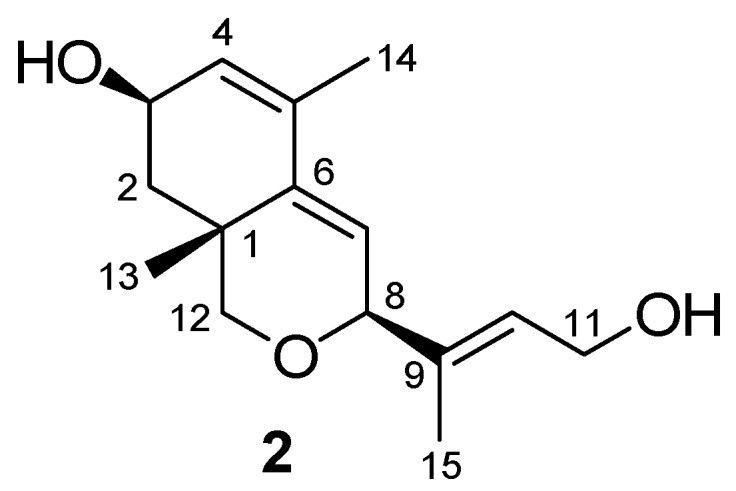
Apocarotenoid **2**.

These and all other data allowed us to establish the structure of **2** as 8,12-epoxy-1,6-cyclofarnesa-4,6,9-triene-3,11-diol (Figure 3), a new C15 apocarotenoid. 

**Figure 4:**
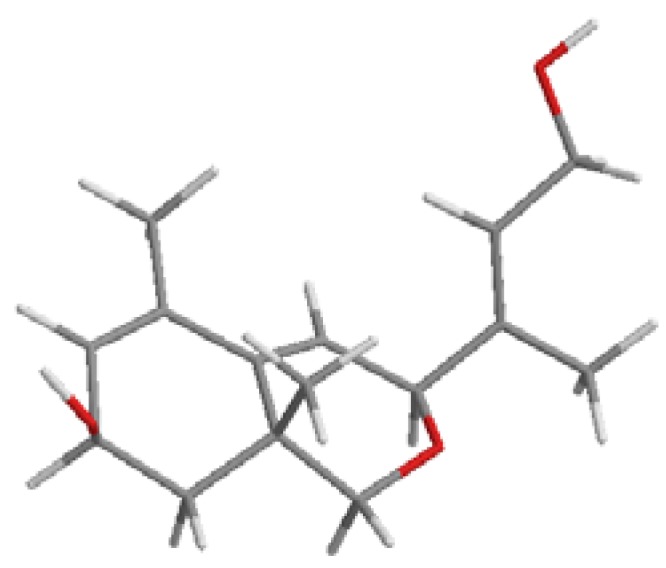
Conformation of apocarotenoid **2**.

The side chain double bond compound possesses the *E* stereochemistry as indicated by the ^13^C-NMR chemical shifts of C11 (δ_C_ 59.5) and C15 (δ_C_ 14.9). Also, relative *syn* stereochemistry between the secondary hydroxyl, the methyl group at C13 and the side chain is proposed based on the following considerations: first, one of the two fused rings, cyclohexene and oxacyclohexene, adopt the most favorable semi-chair conformation depicted in Figure 4 due to the presence of the C13 angular methyl. Secondly, the multiplicity of the H8 (br s) and H3 (br s) in the ^1^H-NMR spectrim involves pseudoaxial and pseudoequatorial dispositions, respectively.

Apocarotenoid **2** is the first compound in *Blakeslea trispora* and the second of all the *Mucoromycotina* fungi that contains a dihydropyran framework. This framework is reminiscent of the azaphylones, fungal metabolites with a polyketide origin [21].

**Scheme 1:**
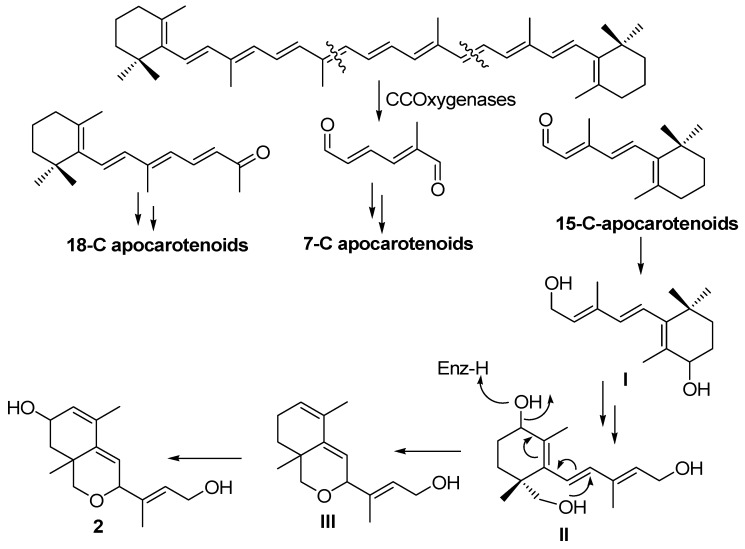
Biosynthetic pathway of apocarotenoid **2**.

Considering its origin (*i.e.*, sexual interaction of *B. trispora*) and its structural framework related to that of C15 apocarotenoids the following biosynthetic pathway is proposed for the formation of **2** (Scheme 1). 

The pathway begins with the double asymmetric β-carotene degradation catalyzed by carotene cleavage oxygenases giving rise to the three fragment precursors of the three families of apocarotenoids (18-C apocarotenoids, 7-C apocarotenoids and 15-C apocarotenoids) [16]. This type of carotene degradation is stimulated by the sexual interaction of opposite sex strains [9,22]. The apocarotenoid **2** comes from the 15 carbons fragment **I**, which undergoes reduction of the aldehyde group to a primary alcohol and then undergoes two hydroxylation processes (mediated probably by cytochrome-P450 dependent enzymes) at C4 and C13 giving rise to the intermediate **II**. At this point a heterocyclization process starting from primary hydroxyl at C13 with a shift of a secondary hydroxyl at C4 by means of a S_N_2' like reaction leads to **III**. Then a new hydroxylation at position C3 on **III** gives rise to metabolite **2**. This biosynthetic pathway suggests the existence of specific hydroxylating enzymes in each *Mucoromicotina* sp. acting at specific positions on each apocarotenoid, and may support the hypothesis of the existence of different sexual signals for each species.

## 3. Experimental

### 3.1. General

NMR spectra (^1^H- and ^13^C-) were recorded with a Varian Direct-Drive 500 (^1^H 500 MHz/^13^C 125 MHz) spectrometer. For high-resolution MS we used an Autospec-Q VG-Analytical (Fisons) mass spectrometer. For semi-preparative normal-phase HPLC the neutral extracts was dissolved in *t*-BuOMe (at 20 g dry extract/L). Aliquots (0.5 mL) were injected onto a column (10 × 250 mm; 5 µm silica particles; Agilent) with a 15 mm refillable guard pre-column filled with the same material placed in a Series 1100 liquid chromatograph (Agilent). The column was eluted at room temperature at a flow rate of 2 mL/min for 25 min with *t*-BuOMe and monitored with a refractometer.

### 3.2. Strains and Culture Conditions

Strains F986 and F921 are wild-type (+) and (−) strains of *Blakeslea* (*Choanephora*) *trispora*, respectively, and were obtained from VKM (All-Russian Collection of Microorganisms, Moscow, Russia). Plates containing 25 mL minimal agar medium [23] were inoculated with 5 × 10^3^ spores of each sex and incubated in the dark at 30 °C for three days.

### 3.3. Extraction and Fractionation of Apocarotenoids.

The initial extracts for apocarotenoid analyses were obtained by freezing (−20 °C for at least 2 h) and thawing (22 °C for 1 h) the media and centrifuging the liquid (4,000 × g, 15 min). Neutral extracts were obtained by adjusting the initial extracts to pH 8.0 with KOH and extracting three times with EtOAc. Acid extracts were obtained by adjusting the remaining aqueous phase to pH 2.0 with HCl and extracting with EtOAc. Water was removed by mixing with anhydrous Na_2_SO_4_ and filtering; the organic solvent was removed by evaporation under low pressure. For the sake of chemical stability, all procedures were carried out under dim light. An initial extract of 500 mL (from 1 L of medium of mated cultures F921 × F986) yielded 114 mg of neutral extract. This neutral extract was fractionated by semi-preparative HPLC. The fraction (16.7 < RT < 17.1 min) contained **2** (4 mg).

*(1R,3R,8S,E)-8,12-Epoxy-1,6-cyclofarnesa-4,6,9-triene-3,11-diol* (**2**): Colourless syrup. [α]_D_ +20.1 (*c* = 1, CHCl_3_). HRMS (FAB), *m/z*: 273.1464 ([M+Na]^+^; calcd. for C_15_H_22_O_3_Na, 273.1467). IR (film) ν_max_: 3417, 2964, 2923, 2857, 1654, 1458, 1407, 1110, 1032 cm^−1^. ^1^H-NMR (CDCl_3_, Me_4_Si): see Table 1. ^13^C-NMR (CDCl_3_, Me_4_Si): see Table 1. 

## 4. Conclusions

The sexual interaction of strains F986 (+) and F921 (−) of *B. trispora* produces known apocarotenoids, in addition to small amounts of a heterocyclic cyclofarnesane whose novel structure corresponds to (3*S*,7*R*,8a*R*)-3-((*E*)-4-hydroxybut-2-en-2-yl)-5,8a-dimethyl-3,7,8,8a-tetrahydro-1*H*-isochromen-7-ol. Biogenetically this apocarotenoid derives from after successive transformations (reduction, regiospecific hydroxylations and heterocyclization) of a 15 carbons fragment produced in a double asymmetric β-carotene degradation. The presence of specific apocarotenoids in each *Mucoromycotina* species reinforces the hypothesis of the existence of different sexual signals. 

## Supplementary Materials

Supplementary materials can be accessed at: http://www.mdpi.com/1420-3049/17/11/12553/s1.
