# Supplementary figures and images for: A Minor Dihydropyran Apocarotenoid from Mated Cultures of Blakeslea trispora
Source: Molecules. 2012 Oct 24;17(11):12553–9. doi: 10.3390/molecules171112553 (PMC6268977; doi:10.3390/molecules171112553)

# Supporting Information

Figure S1. Spectrum  $^1\text{H}$ -NMR of 2.

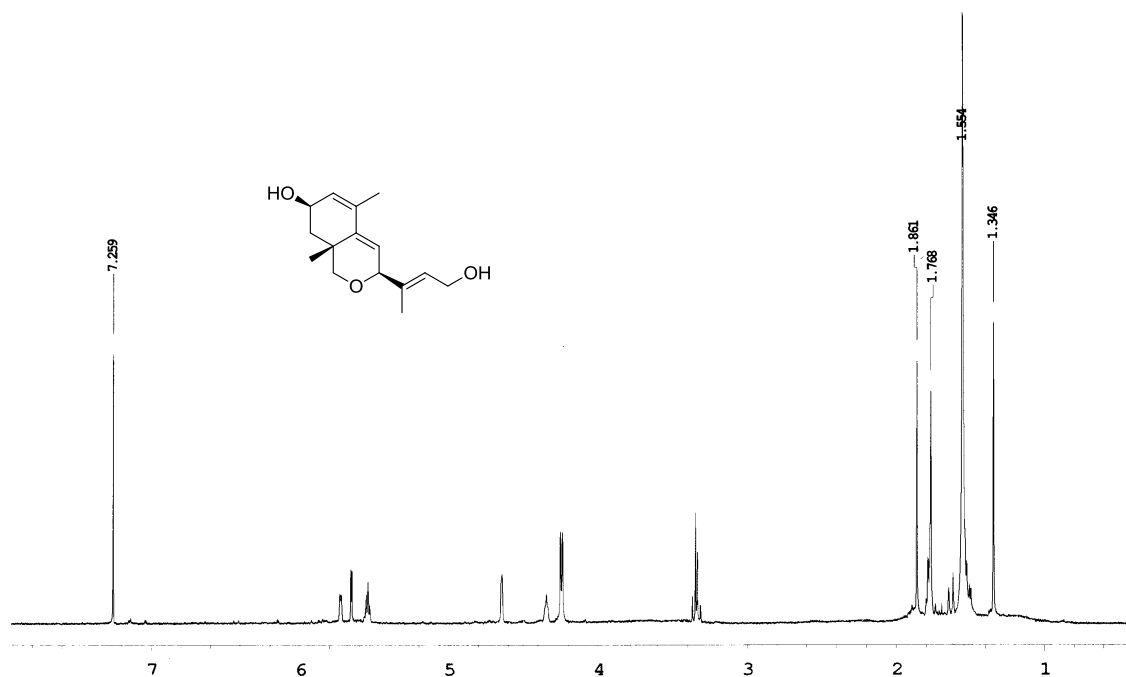

Figure S2. Spectrum  $^{13}\text{C}$ -NMR of 2.

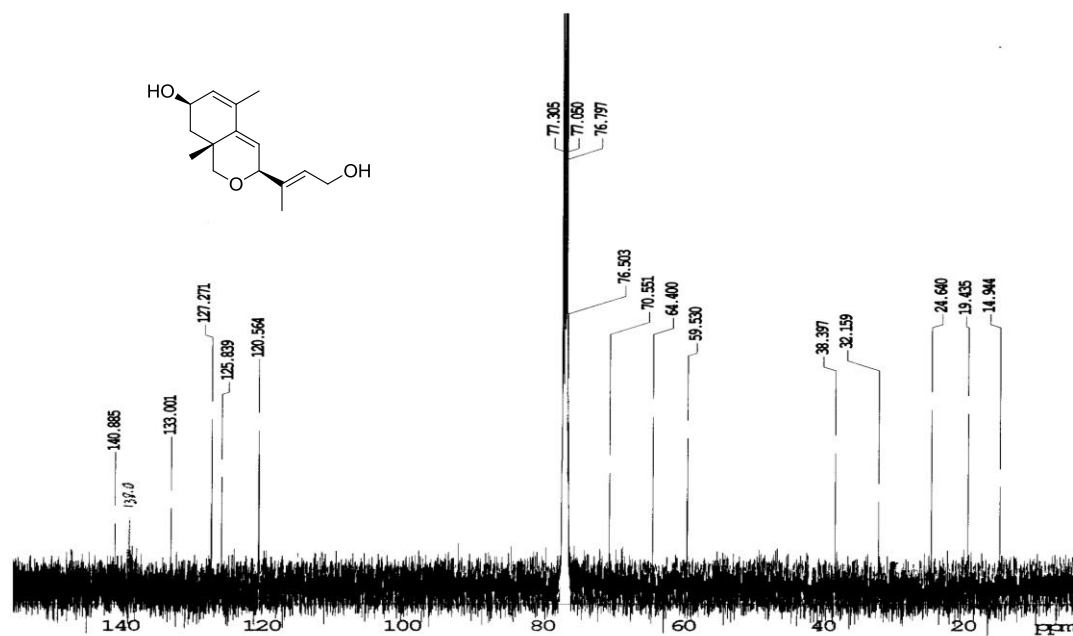

Supplement: Supplementary file 1 [file molecules-17-12553-s001.pdf]
